# Bridging the medical cliff: a paediatric–adult continuity of care model for 2,341 young adults with rare diseases in China

**DOI:** 10.3389/fped.2026.1941475

**Published:** 2026-08-26

**Authors:** Yu Shi, Tianxing Feng, Xuan Gao, Jianshe Wang, Feihong Luo, Fang Liu, Ming Li, Hongsheng Wang, Qian Shen, Hao Li, Shan Zheng, Li Sun, Dahui Wang, Xiaochuan Wang, Libo Wang, Ying Huang, Yuping Qian, Junhua Tian, Ronghua Yang, Jin Fu, Gongbao Liu, Hongquan Geng, Xiaowen Zhai, Yi Wang

**Affiliations:** 1 Outpatient and Emergency Management Office, Children’s Hospital of Fudan University, National Children’s Medical Center, Shanghai, China; 2 Department of Pediatrics, Shanghai Clinical Research and Trial Center, ShanghaiTech University, Shanghai, China; 3 Center for Pediatric Liver Diseases, Children’s Hospital of Fudan University, National Children’s Medical Center, Shanghai, China; 4 Department of Pediatric Endocrinology and Inherited Metabolic Diseases, Children’s Hospital of Fudan University, National Children’s Medical Center, Shanghai, China; 5 Department of Cardiovascular Center, Children’s Hospital of Fudan University, National Children’s Medical Center, Shanghai, China; 6 Department of Dermatology, Children’s Hospital of Fudan University, National Children’s Medical Center, Shanghai, China; 7 Department of Hematology and Oncology, Children’s Hospital of Fudan University, National Children’s Medical Center, Shanghai, China; 8 Department of Nephrology, Children’s Hospital of Fudan University, National Children’s Medical Center, Shanghai Kidney Development and Pediatric Kidney Disease Research Center, Shanghai, China; 9 National Key Laboratory of Kidney Diseases, Shanghai, China; 10 Department of Neurosurgery, Children’s Hospital of Fudan University, National Children’s Medical Center, Shanghai, China; 11 Department of Pediatric Surgery, Children’s Hospital of Fudan University, National Children’s Medical Center, Shanghai, China; 12 Shanghai Key Laboratory of Birth Defect, Key Laboratory of Neonatal Disease, Ministry of Health, Shanghai, China; 13 Department of Rheumatology, Children’s Hospital of Fudan University, National Children’s Medical Center, Shanghai, China; 14 Department of Orthopedics, Children’s Hospital of Fudan University, National Children’s Medical Center, Shanghai, China; 15 Department of Clinical Immunology, Children’s Hospital of Fudan University, National Children’s Medical Center, Shanghai, China; 16 Department of Respiratory Medicine, Children’s Hospital of Fudan University, National Children’s Medical Center, Shanghai, China; 17 Department of Gastroenterology, Pediatric Inflammatory Bowel Disease Research Center, Children’s Hospital of Fudan University, National Children’s Medical Center, Shanghai, China; 18 Department of Medical Affairs, Children’s Hospital of Fudan University, National Children’s Medical Center, Shanghai, China; 19 Department of Urology Surgery, Children’s Hospital of Fudan University, National Children’s Medical Center, Shanghai, China; 20 Department of Neurology, Children’s Hospital of Fudan University, National Children’s Medical Center, Shanghai, China

**Keywords:** China, continuity of care, healthcare delivery, multidisciplinary care, paediatric-to-adult care continuity, rare diseases, young adults

## Abstract

**Introduction:**

Adolescents and young adults with rare diseases face a “medical cliff” when they age out of paediatric services, losing established care relationships and disease-specific expertise. Most rare diseases begin in childhood, since 69.9% of catalogued rare diseases are of exclusively paediatric onset. The problem is acute in China, where an estimated 20 million people live with a rare disease and systematic transitional care pathways remain largely absent.

**Methods:**

We describe the development and implementation of an integrated paediatric–adult continuity of care model for rare diseases, the adult population it serves, and early operational indicators of its feasibility. This retrospective, descriptive analysis used clinical service data from the Children's Hospital of Fudan University, Shanghai, over a 38-month window (3 January 2023–25 February 2026); the model was implemented from September 2023. Its four components were expanded treatment authority with proactive management; a multidisciplinary paediatric–adult joint clinic; proactive follow-up with dynamic evaluation; and medical social work with psychosocial support. The programme admits patients aged 18–35 years.

**Results:**

In total, 2,341 patients aged ≥18 years generated 4,847 outpatient, emergency, and inpatient encounters (mean age at first encounter 22.7 ± 5.9 years; 51.7% female). Annual encounter volumes rose from 240 in 2023 to 1,412 in 2024 and 2,628 in 2025, with a further 567 in January and February 2026. Return attendances accounted for 4,373 encounters (90.2%), and 843 patients (36.0%) attended more than once. The leading specialities were neurology (29.1%), hepatology (12.2%), and endocrinology (8.1%); most encounters were by Shanghai residents (54.8%).

**Discussion:**

Repeat attendance measures utilisation rather than adherence, and rising volumes likely reflect progressive implementation and growing awareness rather than model effectiveness. With enabling policies, paediatric hospitals can deliver continuous care for adult rare disease patients, although these indicators describe service activity rather than clinical or patient-reported outcomes. The model is best characterised as continuity of care delivered within paediatric services rather than as a conventional transition programme, and offers a replicable blueprint for health systems lacking established adult subspeciality services; cross-provincial health insurance portability remains the single most critical barrier to nationwide scale-up.

## 1 Introduction

Rare diseases collectively pose a significant global public health challenge, affecting an estimated 300 million people worldwide (1). Within contemporary healthcare systems, the rigid age-based demarcation between paediatric speciality hospitals and adult medical facilities creates a structural discontinuity that disproportionately affects patients with childhood-onset chronic conditions. As adolescents with rare diseases age out of paediatric services, they often encounter a “medical cliff”, losing the established care relationships, disease-specific expertise, and coordinated management they previously relied upon (2).

Rare diseases are disproportionately conditions of childhood: of the 6,172 rare diseases catalogued in Orphanet, 69.9% are of exclusively paediatric onset (1), so most affected individuals acquire a lifelong condition well before they reach adulthood. The scale of the resulting challenge is particularly acute in China, where an estimated 20 million people live with a rare disease (3). National registry data illustrate both the burden and the difficulty of reaching a diagnosis. Among the first 62,590 cases enrolled in the National Rare Diseases Registry System, the mean age at definitive diagnosis was 30.9 years and 36.1% of registrants were younger than 18 years. Five specialities, namely neurology, endocrinology, haematology, cardiovascular medicine, and nephrology, accounted for 82.0% of registrations (4). Despite this growing population, China's healthcare infrastructure has historically lacked formalised transition pathways, leaving many young adult patients with fragmented medical care, delayed policy coordination, and inadequate resource allocation (3, 5). The consequences include preventable disease progression, increased emergency department utilisation, and substantial psychological distress for patients and families navigating an unprepared system.

Internationally, several models have been developed to address transitional care needs, including dedicated transition coordinators, young adult clinics, and structured referral protocols between paediatric and adult services in North America, Europe, and Australia, most notably the Six Core Elements of Health Care Transition endorsed by North American professional societies (6–9). However, these models were designed within healthcare systems that share three structural advantages: well-established primary-to-tertiary care integration, universal or near-universal health insurance coverage, and mature adult subspeciality services for congenital and childhood-onset conditions. None of the three can be assumed in China's current healthcare landscape.

In recent years, innovative approaches have emerged within China. Paediatric speciality hospitals in Shanghai and Zhejiang Province have pioneered the provision of continuous treatment for rare disease patients aged 18 years and older, departing from the traditional age-restricted model (10). Building upon this foundation, the Children's Hospital of Fudan University, a National Children's Medical Centre, has developed a paediatric–adult continuity of care model for rare diseases. The model shifts from the conventional passive referral paradigm to a proactive, full-cycle management approach coordinated by the original paediatric treatment team.

This study had four objectives. The first was to describe the development, governance, and operational components of an integrated paediatric–adult continuity of care model for rare diseases implemented at a National Children's Medical Centre in China. The second was to assess the feasibility of delivering such care within a paediatric hospital, including the regulatory, institutional, and workforce preparations required. The third was to characterise the adult rare disease population served by the model in terms of demographic profile, geographic origin, and disease spectrum. The fourth was to report early operational indicators of service uptake and continuity of contact over a 38-month observation window. We further examine the physician- and patient-level barriers encountered, contextualised against domestic and international comparators. This study was not designed to evaluate clinical effectiveness; no clinical outcomes, patient-reported outcomes, or comparator group were included.

## 2 Materials and methods

### 2.1 Study design and setting

This was a retrospective, descriptive analysis of clinical service data collected from a National Children's Medical Centre in Shanghai, an 800-bed tertiary paediatric referral hospital serving the Yangtze River Delta region and beyond. The observation window spanned 3 January 2023 to 25 February 2026, a period of 38 months. The integrated paediatric–adult continuity of care model was implemented from September 2023; the 135 encounters recorded between January and August 2023 therefore predate implementation and are retained as a pre-implementation baseline.

In December 2021, a multidisciplinary expert symposium was convened at the hospital, bringing together paediatric subspecialists, adult physicians from comprehensive hospitals, health policy experts, and hospital administrators. The consensus reached at this symposium, which addressed eligibility criteria, clinical governance, legal safeguards, and health insurance coordination, provided the conceptual foundation for the care model subsequently submitted to and approved by the Shanghai Municipal Health Commission.

### 2.2 Development of the paediatric–adult continuity of care model

The care model was designed around four core components, collectively intended to eliminate disciplinary and age-based barriers to continuous care:

(1) Expanded treatment authority and proactive management. The previous institutional age cutoff of 18 years was formally removed for rare disease patients, transitioning the care paradigm from passive, age-driven referrals to a full-cycle proactive management approach. The original paediatric treatment team retained primary responsibility for coordinating all aspects of care, with adult physician consultation invoked on an as-needed basis.
(2) Multidisciplinary collaboration and coordinated co-management. A combined paediatric–adult multidisciplinary clinic (the MDT Bridge Clinic) was established, staffed by paediatric specialists, adult physicians from collaborating institutions, genetic counsellors, and dedicated case managers. Standardised, interoperable electronic health records integrating longitudinal medical history, genetic testing results, and medication reconciliation data were maintained to ensure informational continuity across care settings.
(3) Proactive follow-up and dynamic evaluation. The care team implemented systematic, long-term tracking of each patient's clinical trajectory, actively monitoring for treatment interruptions, missed follow-up appointments, and disease progression events. Automated alerts were triggered when predefined clinical thresholds were breached.
(4) Medical social work and psychosocial support. Social workers from the hospital's Department of Social Work are deployed into clinical teams by ward area and provide individual casework, family emotional support, and facilitation of communication between families and clinicians. They also link families to charitable funding and to accommodation support for those travelling from other provinces, and organise patient mutual-aid and life-education activities intended to alleviate transition-related anxiety, promote self-management, and strengthen psychological resilience.

### 2.3 Study population

All patients aged 18 years and older managed within the paediatric–adult continuity of care programme who received outpatient, emergency, or inpatient services at the hospital during the observation window were included. An upper age limit of 35 years applies to the programme under the regulatory approval granted by the Shanghai Municipal Health Commission. Patients are eligible for continued care within the paediatric institution up to that age and cease to be eligible for the programme on reaching it; the timing of any earlier transfer to adult services is determined on clinical grounds. The First National List of Rare Diseases, issued by the National Health Commission of China together with partner ministries in 2018 (11), and the Second National List, issued in September 2023 (12), constitute the reference framework within which the programme operates and determine eligibility for the associated orphan-drug access and reimbursement provisions. We did not, however, apply the lists as a strict inclusion filter. Their primary function is to govern orphan-drug approval, procurement, and insurance reimbursement rather than to delimit the clinical population of rare disease, and they therefore enumerate a deliberately restricted set of conditions. Many childhood-onset rare and complex conditions managed by the programme are not among them.

The cohort reported here is instead defined by the service itself, and comprises every attendance by a patient aged 18 years and older managed within the programme during the observation window. Data were extracted from the hospital's electronic health record system and included demographic characteristics (age, sex, geographic origin), visit type (outpatient, emergency, or inpatient), clinical department, primary diagnosis, and insurance category. Patients were additionally classified by care origin on the basis of the first-visit/return-visit status recorded at registration. Those with a documented attendance at the institution before 18 years of age, whose care then continued uninterrupted into adulthood, were classified as previously established. Those whose first-ever attendance fell at or after 18 years of age were classified as newly presenting as adults. Two units of analysis are used throughout. The encounter is the unit for service activity indicators, which comprise annual volumes, departmental distribution, geographic origin of attendances, and the return-attendance proportion. The individual patient, identified by a unique hospital record number, is the unit for demographic characteristics, which comprise sex, age, age stratum, and care origin. The denominator is stated wherever a proportion is reported. Geographic origin was classified into three tiers based on distance from Shanghai. Tier 1 comprised neighbouring Yangtze River Delta provinces such as Jiangsu, Zhejiang, and Anhui; Tier 2 medium-to-long-distance provinces such as Henan and Shandong; and Tier 3 more distant provinces such as Sichuan, Beijing, and Shaanxi.

### 2.4 Data extraction, cleaning, and quality control

Records were retrieved from the hospital information system using a predefined extraction template and were assembled into a single analytic file of 4,847 encounter records across ten fields. Completeness was audited field by field. The outpatient record number was missing for one record (0.02%) and the insurance category for four (0.08%); date of attendance, age, sex, department, diagnosis, visit type, and province of residence were complete for every record. All recorded ages were numeric and fell within the eligible range of 18–35 years, and all attendance dates were well formed. Patient names were masked at the point of extraction, so the analytic file contained no directly identifying information. Two residual data quality issues were identified and are reported rather than corrected. First, 58 records were identical across all ten extracted fields; because the registration system permits more than one registration for the same patient, department, and diagnosis on a single day, these could not be distinguished from genuine same-day repeat registrations and were therefore retained. Second, four outpatient record numbers were associated with more than one masked name and two with discordant sex, indicating a small amount of identifier reuse or data-entry error in the source system; together these affected fewer than 0.2% of records and were left unaltered. Diagnoses were recorded as free text at the point of registration, yielding 1,368 distinct diagnostic strings, and were used to characterise the disease spectrum reported in the Results.

A minority of the attendances counted here do not represent care of a patient with a rare disease. A total of 210 encounters (4.3%), contributed by 57 patients (2.4%), were by adult relatives attending as haematopoietic stem cell, liver, or kidney donors for a child treated at the hospital; 189 of these were in the haematology department. A further 211 encounters (4.4%), contributed by 168 patients (7.2%), were stand-alone counselling contacts, predominantly genetic, prenatal, and reproductive counselling, which the programme regards as part of the holistic care of families affected by childhood-onset rare disease. Together, 228 patients (9.7%) had no clinical encounter of their own during the observation window, while the remaining 2,113 (90.3%) did. These attendances have been retained because they form part of the workload the programme actually carries.

### 2.5 Statistical analysis

Descriptive statistics were used to summarise patient characteristics, visit volumes, and departmental distribution. Categorical variables were reported as frequencies and percentages; continuous variables were expressed as mean ± SD. Age at first encounter was additionally summarised in strata of 18–21, 22–25, 26–30, and older than 30 years. All analyses were performed using IBM SPSS Statistics v31.0 (IBM Corp., Armonk, NY, USA). Given the descriptive nature of this analysis, no formal hypothesis testing was performed. No sample size calculation was undertaken, because the entire eligible population treated during the study period was included rather than a sample of it. This manuscript was prepared in accordance with the STROBE reporting guideline for observational studies; the completed STROBE checklist is provided as Supplementary Material.

## 3 Results

### 3.1 Baseline characteristics

A total of 2,341 individual patients aged 18 years and older attended the hospital during the 38-month observation window, generating 4,847 outpatient, emergency, and inpatient encounters, a mean of 2.07 encounters per patient. Of the 2,341 patients, 1,211 (51.7%) were female and 1,130 (48.3%) were male. At encounter level the balance was reversed, with 2,593 encounters (53.5%) by male and 2,254 (46.5%) by female patients, reflecting a higher attendance frequency among male patients. Age at first encounter ranged from 18 to 35 years (mean 22.7 ± 5.9 years; median 19 years). Across all encounters the mean age was 21.4 ± 5.2 years. Stratified by age at first encounter, 1,416 patients (60.5%) were aged 18–21 years, 183 (7.8%) were aged 22–25 years, 336 (14.4%) were aged 26–30 years, and 406 (17.3%) were older than 30 years. With respect to care origin, 1,879 patients (80.3%) had been followed at this institution during childhood and continued their care within the programme after reaching 18 years of age, whereas 462 (19.7%) attended the hospital for the first time as adults.

**Figure 1:**
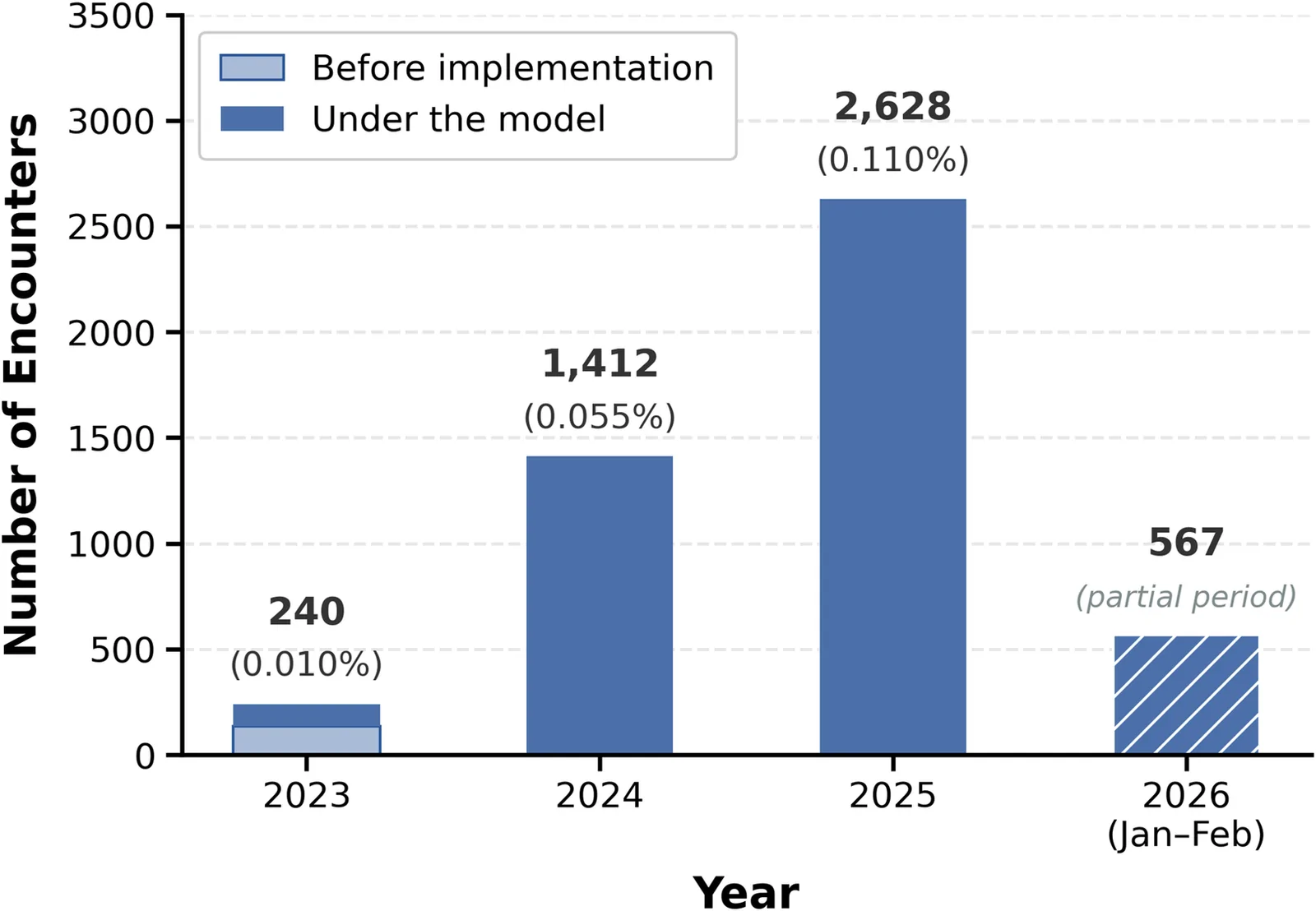
Annual number of encounters by patients aged 18 years and older, January 2023 to February 2026. Bars represent absolute encounter numbers; above each bar the bold figure is the encounter count and the figure in parentheses beneath it is the share of all hospital outpatient and emergency attendances recorded in that period. Within the 2023 bar, the pale segment represents the 135 encounters recorded between January and August 2023, before the model was implemented in September 2023, and the solid segment the 105 encounters recorded thereafter. The hatched bar denotes the partial period January to February 2026; a hospital-wide denominator was not available for that period, so no share is given for it.

Annual encounter volumes increased markedly over the observation window, from 240 in 2023 to 1,412 in 2024 and 2,628 in 2025, with a further 567 encounters recorded in January and February 2026. Of the 240 encounters in 2023, 135 occurred before the model was implemented in September 2023 and 105 thereafter. The proportion of all hospital outpatient and emergency attendances accounted for by this group rose from 0.010% in 2023 (240 of 2,508,270 attendances) to 0.055% in 2024 (1,412 of 2,563,060) and 0.110% in 2025 (2,628 of 2,379,039); a hospital-wide denominator was not available for the partial year 2026. Return attendances accounted for 4,373 of 4,847 encounters (90.2%), and 843 of 2,341 patients (36.0%) attended more than once during the observation window (Figure 1).

**Figure 2:**
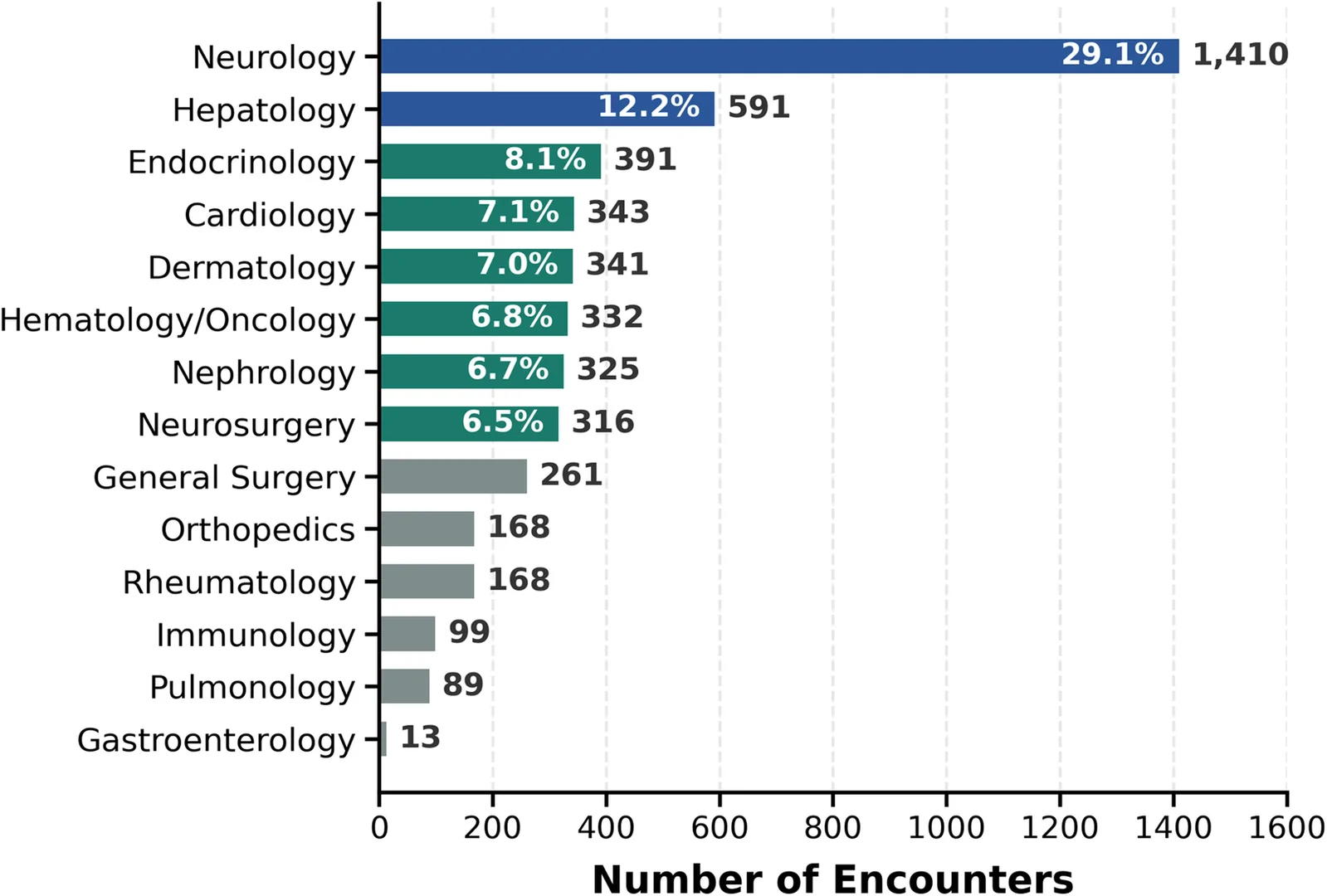
Geographic distribution of encounters by province and municipality of residence (*n* = 4,847). Bars are colour-coded by tier: Shanghai (blue), Tier 1 Yangtze River Delta provinces (teal), Tier 2 medium-distance provinces (orange), and Tier 3 and other distant provinces (grey).

Geographically, most encounters were by residents of Shanghai (*n* = 2,655; 54.8% of encounters, contributed by 1,296 patients, 55.4% of the cohort). Encounter volumes decreased progressively with increasing distance from Shanghai. In Tier 1 (neighbouring Yangtze River Delta provinces), Jiangsu accounted for 820 encounters, Anhui 493, and Zhejiang 382, collectively forming the primary surrounding catchment area. In Tier 2 (medium-to-long-distance provinces), volumes declined substantially, with 122 encounters from Henan and 83 from Shandong. Tier 3 provinces, including Sichuan, Jiangxi, Beijing, and Shaanxi, each accounted for 55 or fewer encounters (Figure 2).

### 3.2 Disease spectrum and departmental distribution

**Figure 3:**
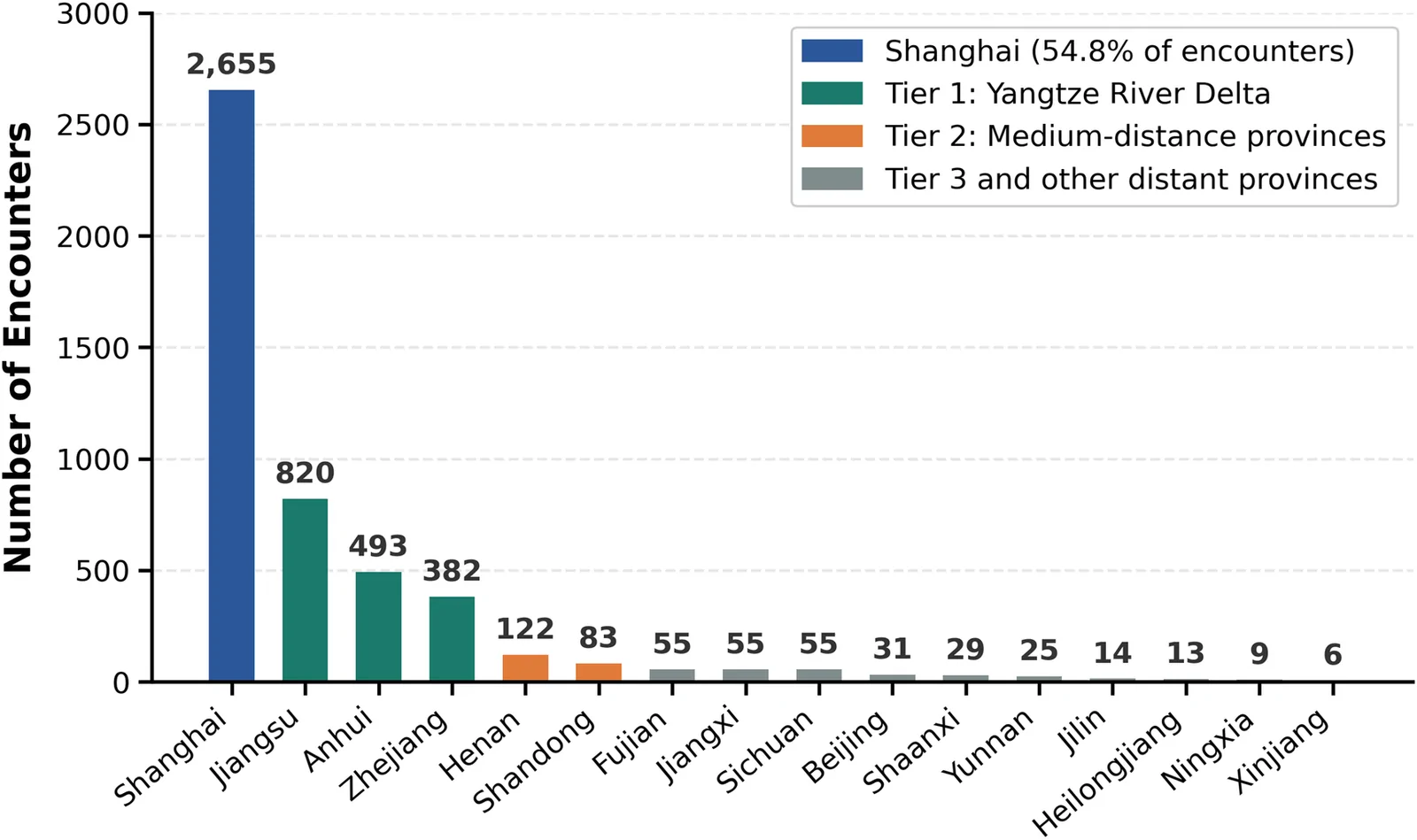
Distribution of encounters by speciality department (*n* = 4,847). Neurology accounted for the largest share (29.1%, *n* = 1,410), followed by Hepatology (12.2%, *n* = 591) and Endocrinology (8.1%, *n* = 391). Percentages are shown for departments accounting for at least 6% of encounters. Departmental categories aggregate the clinic-level codes recorded at registration.

The departments with the highest encounter volumes were Neurology (*n* = 1,410; 29.1% of 4,847 encounters), Hepatology (*n* = 591; 12.2%), and Endocrinology (*n* = 391; 8.1%) (Figure 3). Departmental categories aggregate the clinic-level codes recorded at registration. The Endocrinology category includes 31 clinical genetics attendances; Cardiology combines paediatric cardiology with cardiac surgery; and Haematology and Oncology combines the haematology clinic with oncology medical and surgical attendances. The diseases managed within these departments, including Wilson disease, 21-hydroxylase deficiency, glycogen storage disease, Duchenne muscular dystrophy, and haemophilia, are representative of the broader category of rare diseases requiring lifelong, multi-system management (Table 1). The next largest volumes were in Haematology and Oncology, Nephrology, Cardiology, Rheumatology, and Immunology.

**Table 1:** Departments and representative rare diseases managed under the continuity of care model.

Department	Representative rare diseases
Neurology	Angelman syndrome, congenital myasthenic syndrome, generalised myasthenia gravis, Huntington disease
Hepatology	Wilson disease, progressive familial intrahepatic cholestasis
Endocrinology	21-hydroxylase deficiency, congenital adrenal hypoplasia, glutaric acidaemia type I, phenylketonuria, metachromatic leucodystrophy
Cardiology	Glycogen storage disease (type I, type II), idiopathic cardiomyopathy, methylmalonic acidaemia
Dermatology	Albinism, hidradenitis suppurativa
Haematology and Oncology	Haemophilia, Langerhans cell histiocytosis, familial haemophagocytic lymphohistiocytosis, thalassaemia major, neuroblastoma
Nephrology	Alport syndrome, Fabry disease, Gitelman syndrome, haemolytic uraemic syndrome, thrombotic thrombocytopenic purpura
Neurosurgery	Glioblastoma
General Surgery	Biliary atresia, primary biliary cholangitis
Rheumatology	Systemic sclerosis, ANCA-associated vasculitis, Behçet disease, systemic juvenile idiopathic arthritis, Takayasu arteritis
Orthopaedics	Congenital scoliosis, fibrodysplasia ossificans progressiva
Immunology	Primary combined immunodeficiency, X-linked agammaglobulinaemia, X-linked lymphoproliferative disease
Pulmonology	Cystic fibrosis, idiopathic pulmonary fibrosis, pulmonary alveolar proteinosis
Gastroenterology	Inflammatory bowel disease

## 4 Discussion

This study presents the first comprehensive account in China of the practical approaches, early operational indicators, and implementation challenges associated with an integrated paediatric–adult continuity of care model for rare diseases. Our findings indicate that, with enabling policy frameworks, organisational restructuring, and innovative service design, paediatric speciality hospitals can legally and clinically transcend traditional age restrictions to deliver continuous treatment for adult rare disease patients, thereby helping to mitigate the medical cliff phenomenon.

Three findings frame the discussion that follows. First, the service reached scale quickly, with annual encounter volumes rising from 240 in 2023 to 2,628 in 2025 and a further 567 in the first two months of 2026. Second, the programme is predominantly retaining its own patients rather than recruiting adults *de novo*: 1,879 of 2,341 patients (80.3%) had been followed at this institution during childhood, and 4,373 of 4,847 encounters (90.2%) were return attendances. Third, the population served is concentrated in a small number of specialities, principally neurology, hepatology, and endocrinology, and geographically in Shanghai and the surrounding Yangtze River Delta, with attendance falling steeply with distance. This activity followed a shift from passive, age-driven referral to prospective management coordinated by the established paediatric care team. We consider in turn the feasibility of the model and how it differs from international transition programmes, the age boundary and the criteria for transfer, and the physician- and patient-level barriers encountered. We then turn to the institutional preparation and workforce implications of delivering adult care in a paediatric hospital, the disease spectrum and the wider life-course needs it generates, and the potential disadvantages of the approach.

### 4.1 Feasibility and international comparisons

The primary prerequisite for paediatric hospitals to extend care into adulthood is the removal of legal restrictions on paediatric scope of practice. Within Shanghai, the Shanghai Children's Medical Centre has concurrently explored transitional pathways for adolescent and young adult patients, particularly in cardiology and haematology/oncology (10). Both institutions participated in the 2021 expert symposium and agreed that paediatric hospitals can serve as medical homes for young adults with childhood-onset rare diseases, provided that structured referral protocols to adult services exist for conditions beyond paediatric expertise. In Zhejiang Province, a children's hospital obtained provincial approval to admit patients aged 18 years and older, a precedent that directly informed the Shanghai model.

Internationally, the model differs from approaches reported in Western healthcare systems, where transitional care has largely been operationalised through transition coordinator roles, young adult clinics, and structured readiness assessments (6–9, 13). These models emphasise patient education and the gradual transfer of responsibility, and they presuppose well-resourced adult subspeciality services capable of receiving the patients transferred. That presupposition does not hold for many rare diseases in China, where adult physicians frequently lack training in conditions presenting predominantly in childhood. Boston Children's Hospital, the Hospital for Sick Children in Toronto, and Great Ormond Street Hospital already care for adults with childhood-onset conditions through adolescent and young adult medicine programmes; the Chinese model shares that logic but operates in a very different regulatory and financing environment.

Most internationally accepted transition programmes, including those structured around the Six Core Elements of Health Care Transition, are designed to prepare young people for, and ultimately to effect, a transfer of care to adult providers (9). The model described here does not do this. Patients remain within the paediatric institution under the continuing responsibility of their original subspecialty team, with adult physicians contributing in a consultative and co-management capacity rather than assuming primary responsibility. It is therefore more accurately described as continuity of care delivered within paediatric services than as a healthcare transition programme in the conventional sense. The two approaches address the same underlying problem, the loss of expert care at the age boundary, but resolve it in opposite directions. Which is appropriate depends largely on whether the health system has adult services able to receive these patients (14).

### 4.2 The age boundary and criteria for transfer

The programme operates to an upper age limit that is set externally rather than left to clinical discretion. The regulatory approval granted by the Shanghai Municipal Health Commission permits continued care within the paediatric institution up to 35 years of age, at which point patients cease to be eligible for the programme. Within that ceiling the timing of transfer is a clinical judgement. In practice it is considered when three conditions converge. The patient's condition must be clinically stable on a maintenance regimen; a competent adult service for the specific condition must be accessible to the patient, including under their insurance arrangements; and age-specific adult health needs, such as cardiovascular risk management, cancer screening, pregnancy and reproductive care, or the management of acquired adult comorbidity, must begin to outweigh the benefit of continued paediatric subspecialty oversight.

Under a conventional age cutoff, a young person must identify and enter an unfamiliar adult service at the moment of legal adulthood, which is also the moment they are leaving school, entering work or higher education, and taking responsibility for their own health for the first time. Extending the boundary to 35 years converts that abrupt requirement into a long buffer during which the patient can find adult care that suits them, at a pace of their own choosing, with the paediatric team still in place while they do so. Codifying graduated transfer criteria within the 18-to-35-year window is a priority for the next phase of the programme.

### 4.3 Physician-level barriers

The successful implementation of this model required overcoming deeply entrenched physician-level barriers. A fundamental shift in clinical mindset was necessary. Paediatricians had long been trained within an age-bounded professional identity, and extending care to adult patients required expanding clinical scope to encompass adult physiology, pharmacology, and disease manifestations. Physician acceptance was built through departmental discussion, patient narratives illustrating the harms of care disruption, and consensus-building around the ethical obligation to provide continuity for patients with lifelong conditions.

A more difficult barrier than clinical mindset is uncertainty about the timing of referral. For most childhood-onset rare diseases there is no evidence indicating when continued paediatric follow-up ceases to serve a patient better than adult care would, no established indicator marking the point of transfer, and no feedback from adult services on how transferred patients fare. Decisions consequently vary between clinicians faced with similar patients, and transfer tends to be prompted by an event rather than planned. Converting the criteria described above into an explicit and reviewable schedule remains a work in progress.

Paediatricians in our institution reported limited experience in managing adult-specific complications of childhood-onset diseases. A salient example involves premature coronary atherosclerosis arising from longstanding dyslipidaemia in patients with inherited metabolic disorders. In such cases, close collaboration with adult cardiologists was essential, underscoring the critical importance of the MDT Bridge Clinic mechanism. Similarly, age-appropriate cancer screening, management of osteoporosis, and sexual and reproductive health counselling represent areas requiring input from adult-medicine colleagues.

Legal and regulatory frameworks had to be navigated carefully. Chinese law has historically restricted paediatricians’ scope of practice to patients under 18 years of age. The Shanghai Municipal Health Commission's approval represented an administratively significant unbinding of the hospital from strict age limitations, providing legal protection to participating physicians and establishing a regulatory precedent. Nevertheless, the absence of a national-level statutory framework for transitional care leaves physicians in other regions without comparable safeguards, representing a structural barrier to nationwide scale-up.

### 4.4 Patient-level barriers

From the patient perspective, psychological acceptance of continued treatment at a children's hospital was not uniform among adult patients. Some expressed discomfort with the paediatric care environment, including waiting rooms populated predominantly by young children, age-inappropriate decor, and inpatient wards that did not separate patients by sex. In response, the hospital has begun designating dedicated clinic sessions for adolescent and adult patients and is planning the renovation of age-appropriate inpatient units.

The most consequential structural barrier concerns health insurance coverage. At present, only a limited number of provinces and municipalities, including Shanghai and Zhejiang, permit local health insurance to reimburse paediatric hospital services for patients aged 18 years and older with qualifying rare diseases. Because the hospital serves as a national referral centre, a substantial proportion of its patient population travels from other provinces. For these patients, cross-provincial health insurance does not currently cover services received at a paediatric hospital beyond age 18, creating a severe financial disincentive to continuity of care. Resolving this inter-provincial insurance portability gap is the single most urgent policy priority for scaling the model nationally.

Additional patient-level barriers include low health literacy regarding the rationale for lifelong speciality follow-up and logistical obstacles such as transportation costs and lost wages associated with long-distance travel. These barriers are reflected in the sharp drop-off in patient numbers from Tier 2 and Tier 3 provinces.

### 4.5 Institutional preparation, workforce, and sustainability

Extending paediatric services to adult patients required substantial institutional preparation. Medical equipment, including blood pressure cuffs, intravenous access devices, and imaging-compatible accessories, was updated to accommodate adult body habitus. The hospital pharmacy expanded its formulary to include adult-appropriate medication dosages and formulations. Consumables and disposable supplies were reviewed and replaced where paediatric-specific products were unsuitable for adult patients.

The clinical laboratory established adult reference ranges for all routine biochemical, haematological, and endocrine assays, a foundational requirement for safe clinical decision-making in adult patients. The radiology department implemented adult-appropriate radiation dose protocols for computed tomography and plain radiography, as paediatric dose-reduction strategies may compromise diagnostic image quality in adult patients.

Workforce preparation is a parallel and more demanding requirement. Paediatric subspecialists are being asked to acquire and maintain competence in the adult manifestations of childhood-onset disease alongside an undiminished paediatric workload. In the short term this has been absorbed through the MDT Bridge Clinic, which concentrates adult expertise where it is most needed. Three measures appear necessary over the medium term. Adult continuity work should be formally recognised in job planning and workload accounting. Structured continuing education in adult medicine should be provided, ideally jointly with partner adult institutions. Finally, clinicians with dual paediatric and adult training should be appointed, or adult physicians embedded within the paediatric service.

Because patients cease to be eligible at 35 years, the adult population served does not accumulate without limit, and the caseload will approach a steady state once the earliest cohorts reach the ceiling, set thereafter by the annual number of patients turning 18 and by their average duration within the programme. The current phase is the demanding one. Entries greatly exceed exits, and the institution must absorb a caseload that grows year on year using a workforce, an estate, and a funding stream that were all designed for children.

### 4.6 Disease spectrum and holistic care needs

The disease spectrum observed in our cohort, with neurology, hepatology, and endocrinology predominating, is consistent with the recognised distribution of childhood-onset rare diseases that require lifelong, multi-system specialist management (14), for which genomic and epigenomic characterisation continues to evolve (15). The same three specialities dominate national registry data, in which 82.0% of registered rare disease cases in China come from neurology, endocrinology, haematology, cardiovascular medicine, and nephrology (4). Conditions such as Wilson disease, 21-hydroxylase deficiency, glycogen storage disease, and haemophilia are typically diagnosed in childhood but continue to present evolving challenges in adulthood, including multi-organ complications, fertility and reproductive concerns, genetic counselling needs, and psychological adaptation to chronic illness.

Our findings highlight that treatment continuity for patients in the 18–35 year age range began to encompass fertility counselling, genetic risk evaluation for offspring, workplace accommodation, and psychological support (16), reflecting a holistic approach extending beyond disease management to broader life-course needs. Evidence indicates that anxiety and depression prevalence among young adults transitioning with rare diseases is significantly elevated relative to the general population (17), and the medical social work component is directed at improving self-management capacity and psychological resilience. The accurate identification of these heterogeneous conditions is further underpinned by the hospital's established multidisciplinary team programme for rare and undiagnosed diseases (18).

### 4.7 Potential disadvantages and unintended consequences

A balanced appraisal must weigh the costs of this approach alongside its benefits. The most important is that retaining patients within paediatric services may delay their integration into the adult healthcare system. The extended ceiling is intended to make that delay productive rather than a simple postponement, but the risk remains that the preparation does not in fact take place, and a patient who reaches 35 years without having engaged with adult services faces a more abrupt discontinuity than one transferred at 20 with support. Prolonged care within a familiar paediatric team may also reinforce dependence on individual clinicians and sustain parental involvement in medical decision-making beyond the point at which it serves the patient, at the expense of the self-advocacy and system-navigation skills that adult healthcare demands. The medical social work component of our model was designed in part to counter this, but we have not measured whether it does so.

There are clinical risks as well. Paediatric institutions have limited routine exposure to adult-onset comorbidity, and several of the conditions that will determine long-term outcomes for this population, including hypertension, dyslipidaemia, malignancy, osteoporosis, and reproductive and mental health needs, fall outside customary paediatric practice. The MDT Bridge Clinic mitigates this but does not eliminate it, and the residual risk grows with the age of the population served. Patient discomfort with the paediatric care environment is likewise probably understated, because patient experience was not collected systematically. None of these considerations argues against the model in the current Chinese context, but together they define the conditions under which it should continue to be regarded as adequate.

### 4.8 Limitations

This study has several limitations. First, the data come from a single paediatric speciality hospital, which may limit generalisability. Second, the 38-month observation window, of which 30 months follow implementation, is short; because rare diseases require lifelong management, five to ten years of follow-up would be needed to establish whether the continuity of contact achieved here translates into differences in disease progression, complication rates, or survival. Third, no comparison group was included, limiting causal inference. Service growth coincided with programme rollout and increased awareness, making implementation reach and patient uptake at least as plausible explanations as model effectiveness. Fourth, patient volumes, visit patterns, and repeat attendance measure service activity, not clinical outcomes, and should not be taken as evidence of clinical benefit. Patient-reported outcomes were not collected either, so although the model incorporates psychosocial support, medical social work, and multidisciplinary coordination, we cannot demonstrate that its patient-centred components achieved their intended effect. Fifth, the electronic record captures non-attendance but not its reasons, so patients who disengaged cannot be distinguished from those who transferred elsewhere, relocated, or died. Sixth, as a retrospective analysis of routinely collected data, the study carries the biases inherent in that design. The cohort is defined by the programme itself rather than by the national rare disease lists, so it includes donor and counselling visits, meaning comparisons with list-defined cohorts should be made cautiously. In addition, patients who had stopped attending before January 2023 are absent from the dataset, which biases estimates of continuity upwards. Future studies should address these issues by using multicentre designs, longer follow-up periods, patient-reported outcome measures including validated transition readiness and quality-of-life instruments, and qualitative methods.

## 5 Conclusion

This study shows that paediatric speciality hospitals in China can, with appropriate policy support, organisational commitment, and infrastructural investment, feasibly deliver continuous care for adult patients with rare diseases. The model, defined by proactive coordination, multidisciplinary collaboration, and comprehensive lifecycle management, achieved a return-attendance rate of 90.2% and rapid uptake, with annual encounters rising from 240 in 2023 to 2,628 in 2025. These are indicators of service uptake and continuity of contact, and the descriptive design does not permit conclusions about clinical effectiveness. The model is best understood as continuity of care delivered within paediatric services rather than as a conventional transition programme. We also regard it as a permanent element of the care pathway rather than a temporary expedient. The question it answers is who holds continuing responsibility for a person with a childhood-onset rare disease, and that question does not expire when adult provision improves, since for many of these conditions the accumulated knowledge of the original subspecialty team is not readily transferable to a service that encounters them rarely (14). It follows that the model should be judged by whether disease is well controlled, whether complications are prevented or detected early, and whether patients live well. Critical challenges for nationwide scale-up include establishing national-level legal frameworks authorising transitional care, building physician competency in adult disease management through MDT mechanisms, and expanding cross-provincial health insurance coverage to eliminate financial barriers to continuity of care. Alongside these, the programme must translate the regulatory 35-year ceiling into graduated transfer criteria backed by a guaranteed receiving service, plan for the workforce and capacity consequences of the ramp-up phase, and evaluate itself against patient-reported and clinical outcomes rather than service activity alone.

## Data Availability

The data analyzed in this study is subject to the following licenses/restrictions: The de-identified dataset analysed during the current study is available from the corresponding authors on reasonable request, subject to institutional data governance approval. Requests to access these datasets should be directed to Xiaowen Zhai, xwzhai@fudan.edu.cn.
